# Fee Bill

**Published:** 1855-09

**Authors:** 


*Fee Bill.*—The following rate of charges or *fee bill*, having reference simply to the more common items of practice, was agreed upon by the Cook County Medical Society in April last, and it has since been approved and signed by nearly all of the active Practitioners in the city. It is an advance of about fifty per cent. on the former rates of charging; and is by no means equal to the advance in the expense of living in this city. Dur-the last three years, for instance, both house-rent and house-keeping have doubled; and the prices of almost everything else have increased in like proportion. Under such circumstances, at the former rate of charging, it was found that it required all the proceeds of a full and laborious practice to give the physician and his family a comfortable support, without accumulating for him fifty dollars annually. Hence the profession of the city has done only a simple act of justice to its own faithful and hard-working members by adopting the rates that follow, viz.:

| | | | |
|---|---|---|---|
| 1st. | | For ordinary visits in the day-time, - - | $1,50 |
| 2d. | " | Night visits, (from 10 o'clock, P.M., to 6 A.M.) | 3,00 |
| 3d. | " | Consultation visit, from - - | 5,00 to 15,00 |
| 4th. | " | Subsequent Consultation, visit to same patient, - . - - - | 2,00 to 3,00 |
| 5th. | " | Physical Examination, chest, &c., from | 3,00 to 5,00 |
| 6th. | " | Attendance on Obstetrical cases, " | 10,00 to 20,00 |
| | " | Use of Instruments in addition, " | 5,00 to 20,00 |
| 7th. | " | Vaccination in the office, - - - | 1,00 |
| | " | do. at the patient's residence in addition to the usual charge for visit 50 cts. for each person vaccinated. | |
| 8th. | " | Attendance on Small Pox cases per visit, | 2,00 |
| 9th. | " | Visits in the country by private conveyance, per mile, | 1,00 |

